# Aetiological Fraction of Influenza, Respiratory Syncytial Virus and Other Respiratory Pathogens in Infants Aged < 1 Year Hospitalised With Respiratory and Non‐Respiratory Medical Illness in South Africa, 2016–2018

**DOI:** 10.1111/irv.70135

**Published:** 2025-07-01

**Authors:** Nicole Wolter, Cheryl Cohen, Anne von Gottberg, Stefano Tempia, Jocelyn Moyes, Claire von Mollendorf, Florette K. Treurnicht, Orienka Hellferscee, Kathleen Subramoney, Malefu Moleleki, Cayla Reddy, Lorens Maake, Mvuyo Makhasi, Neydis Baute, Sibongile Walaza

**Affiliations:** ^1^ Centre for Respiratory Diseases and Meningitis National Institute for Communicable Diseases of the National Health Laboratory Service Johannesburg South Africa; ^2^ School of Pathology, Faculty of Health Sciences University of the Witwatersrand Johannesburg South Africa; ^3^ School of Public Health, Faculty of Health Sciences University of the Witwatersrand Johannesburg South Africa; ^4^ Department of Paediatrics Mapulaneng Hospital Mapumalanga South Africa

**Keywords:** infants, influenza, pneumonia, RSV, South Africa

## Abstract

**Background:**

Understanding the contribution of pathogens to respiratory illness in infants is important to guide interventions. We assessed the aetiology of respiratory pathogens among infants hospitalised with respiratory and non‐respiratory illness.

**Methods:**

We conducted an unmatched case–control study among infants aged < 1 year. Cases were admitted with acute respiratory and non‐respiratory illness in November 2016–October 2018. Controls were infants presenting for immunisation with no reported illness. Nasopharyngeal aspirates and blood were tested using multi‐pathogen real‐time PCR. Aetiological fraction (AF) was calculated using logistic regression, adjusting for HIV, age, season and pathogens with higher prevalence in cases than controls. Factors associated with respiratory illness hospitalisation were assessed using logistic regression.

**Results:**

Overall, 1214 cases (846 respiratory, 368 non‐respiratory) and 684 controls were included. Respiratory syncytial virus (RSV) (AF 94.0%), influenza (AF 72.6%) and human metapneumovirus (HMPV) (AF 74.9%) were significantly attributable to respiratory illness hospitalisation. *Klebsiella pneumoniae* had significant AF in both respiratory (AF 48.0%) and non‐respiratory (AF 60.7%) hospitalisation. HIV exposure (adjusted odds ratio [aOR] 1.5, 95% confidence interval [CI] 1.1–2.0) and living with HIV (aOR 6.6, 95%CI 2.1–20.5), underlying illness (aOR 4.8, 95%CI 1.3–17.6), malnutrition (aOR 6.0, 95%CI 4.0–8.9), infection with RSV (aOR 19.7, 95%CI 11.4–34.1), influenza (aOR 5.7, 95%CI 2.3–14.1) or HMPV (aOR 4.1, 95%CI 2.0–8.6) were associated with respiratory illness hospitalisation.

**Conclusions:**

Maternal immunisation to prevent severe RSV and influenza illness in infants should be prioritised. In addition, improved infant nutrition and the prevention of HIV‐infection and HIV‐exposure could reduce the high burden of severe respiratory illness.

## 1 Introduction

Despite significant progress in reducing childhood mortality, lower respiratory tract infections, such as bacterial and viral pneumonia, bronchitis and bronchiolitis, remain the largest infectious cause of death in children aged < 5 years globally, with the highest number of deaths occurring in sub‐Saharan Africa [1]. Although underlying causes are multi‐factorial, understanding the contribution of different pathogens to the burden of lower respiratory tract infections remains important to guide treatment and intervention strategies.

Several multi‐country studies using comprehensive sampling have helped to elucidate the causative pathogens of severe and fatal pneumonia in children. The Child Health and Mortality Prevention Surveillance (CHAMPS) programme conducted in 2016–2022 found that pneumonia accounted for 40% of all childhood deaths, with the leading bacterial causes of death in children aged 1–59 months with community‐acquired pneumonia being *Streptococcus pneumoniae* and *Klebsiella pneumoniae,* whereas leading viral causes identified were cytomegalovirus and respiratory syncytial virus (RSV) [2]. In the Pneumonia Etiology Research for Child Health (PERCH) case–control study among children aged 1–59 months hospitalised with pneumonia, RSV was found to be the dominant pathogen, with an aetiological fraction of 31% [3]. Other common pathogens detected included rhinovirus, human metapneumovirus (HMPV), parainfluenza virus, *S. pneumoniae* , *Mycobacterium tuberculosis* and *Haemophilus influenzae* . The Etiology of Pneumonia in the Community (EPIC) population‐based surveillance study in the United States showed the highest incidence of pneumonia‐related hospitalisation in children aged < 2 years, with RSV being the most common pathogen detected [4].

Interventions to prevent severe RSV disease in young infants, such as maternal immunisation and long‐acting monoclonal antibodies, have been recently introduced in mostly high‐income countries [5, 6]. However, due to limited resources and competing health priorities, the introduction of RSV interventions in low‐ and middle‐income countries may be delayed or only offered to limited groups.

Estimates of the burden of RSV‐associated severe illness in South Africa in 2011–2016 showed an incidence rate of 6995 (95% confidence interval [CI] 5002–9421) per 100,000 in children aged < 1 year; highest in infants aged < 1 month at 14674 per 100,000 [7]. However, estimates of the burden of RSV and other aetiological causes of pneumonia are commonly restricted to patients with respiratory symptoms and may therefore underestimate the true burden. We assessed pathogen prevalence and aetiological fraction (AF) among hospitalised infants presenting with respiratory and non‐respiratory medical illness to better understand the burden of RSV and other respiratory pathogens in this age group.

## 2 Methods

### 2.1 Study Description

We conducted an unmatched case–control study at three sentinel sites (including a hospital and primary healthcare clinic at each site) in three provinces (KwaZulu‐Natal, Mpumalanga and North West) from November 2016 through October 2018. Surveillance officers enrolled cases (infants aged < 1 year admitted to the medical ward or intensive care unit, with respiratory and non‐respiratory medical illness) and controls. Controls were infants aged < 1 year residing in the hospital catchment area and presenting for immunisation, and whose parents/caregivers reported no signs or symptoms of any illness in the past 14 days including fever, cough, runny nose or diarrhoea for the infant and household members. At enrollment, surveillance officers collected a nasopharyngeal aspirate, whole blood and serum specimens, along with demographic and clinical information obtained by parent/caregiver structured interviews, and hospital record review. Nasopharyngeal aspirates have been shown to have similar sensitivity to nasopharyngeal swabs for the detection of respiratory viruses [8]. A single physical contact follow‐up visit was conducted for cases and controls between 2 and 6 weeks post‐enrollment during which a second serum specimen was collected, and controls were asked whether they had developed symptoms. Controls were excluded if they reported having experienced any respiratory or non‐respiratory symptoms within 14 days of enrollment. Cases were followed up for outcome until discharge or in‐hospital death. Cases were classified as respiratory cases if they met the pneumonia surveillance programme case definition (children aged 2 days to < 3 months with diagnosis of suspected sepsis or physician‐diagnosed acute lower respiratory tract infection (LRTI), and children aged 3 months to < 5 years with physician‐diagnosed LRTI, including bronchitis, bronchiolitis, pneumonia, and pleural effusion) [9], or were classified as non‐respiratory cases if they did not meet the surveillance case definition.

### 2.2 Sample Size

For 80% power and a 95% confidence interval (CI), we aimed to enroll a minimum of 764 cases and 764 controls to detect a significant AF for influenza virus detection, assuming a 0.6% influenza detection rate among controls and a 2.5% detection rate among cases. To achieve the target number of controls, each week at each of the three sites, we aimed to enroll three asymptomatic children: one aged 0–2 months, one aged 3–5 months and one aged 6 months to < 1 year.

### 2.3 Laboratory Testing

Total nucleic acids were extracted from nasopharyngeal and blood specimens using the Roche MagNA Pure 96 instrument (Roche Diagnostics, Mannheim, Germany). Nasopharyngeal specimens were tested using the Fast Track Diagnostics (FTD, Luxembourg) respiratory pathogen 33 panel (detecting *Chlamydia pneumoniae, Staphylococcus aureus, S. pneumoniae, H. influenzae, Moraxella catarrhalis, Mycoplasma pneumoniae, Legionella* spp., *H. influenzae* type b*, Bordetella pertussis*, *Salmonella* spp., *K. pneumoniae* , influenza A/B/C, RSV A/B, HMPV A/B, rhinovirus, human coronaviruses (229E, OC43, NL63, HKU1), human bocavirus, adenovirus, enterovirus, parainfluenza virus (types 1, 2, 3 and 4), *Pneumocystis jirovecii*). Whole blood specimens were tested for *S. aureus, S. pneumoniae, H. influenzae*, *K. pneumoniae* , *Pseudomonas aeruginosa* , Group B Streptococcus (GBS), *Escherichia coli, Listeria monocytogenes, Chlamydia trachomatis, Ureaplasma urealyticum/parvum* and human cytomegalovirus using FTD bacterial pneumonia hospital‐acquired pneumonia (HAP), FTD bacterial meningitis and FTD neonatal sepsis panels (Fast Track Diagnostics).

Serum samples were tested for antibodies against influenza viruses (A(H1N1)pdm09, A(H3N2), B/Victoria and B/Yamagata) representative of lineages circulating at the time of the study using the haemagglutination inhibition (HAI) assay described by the World Health Organization [10]. A fourfold rise in geometric mean titres (GMT) between the enrollment and follow‐up sera was considered recent influenza infection. Serological attack rate was calculated as the number of infants that seroconverted/number of infants with paired sera tested. Infants with a HAI titres > 320 at enrollment, and infants with < 14 days between paired sera collection were excluded from the serological attack rate calculation.

### 2.4 Definition of HIV Status

Infant HIV status was determined from standard of care testing, or through testing at enrollment by polymerase chain reaction (PCR). HIV‐unexposed uninfected (HUU) infants were infants with a negative HIV result and a recently documented (< 3 months) negative maternal HIV status. HIV‐exposed uninfected (HEU) infants were infants with a negative HIV result and a recently documented or verbally reported positive maternal HIV status, evidence that the mother was taking antiretroviral treatment (ART) during pregnancy or post‐partum. Infants living with HIV (ILWH) were infants with a recently documented positive HIV result, verbally reported by the parent/caregiver, or evidence that the infant was receiving ART. If the mother's HIV status was unknown or a negative test result was from > 3 months prior, the mother was offered voluntary counselling and testing.

### 2.5 Statistical Analysis

The PCR detection rate (number of positives/number tested) of each pathogen was compared between cases (all cases, respiratory cases and non‐respiratory cases respectively) and controls using the chi‐squared test. Pathogen detection rates were analysed separately for nasopharyngeal specimens and blood specimens. AF and AF‐adjusted prevalence were calculated for pathogens where the detection rate was significantly higher (*p* < 0.002 for nasopharyngeal specimens [*n* = 30 pairwise comparisons] and *p* < 0.005 for blood specimens [*n* = 11 pairwise comparisons] after Bonferroni correction) for cases compared to controls on the same specimen type. Unconditional random effects logistic regression, accounting for site‐specific clustering, was used to estimate the AF by comparing the detection rate of cases to controls. Estimates were adjusted for HIV status, age group, season (Dec–Feb, summer; Mar–May, autumn; Jun–Aug, winter; Sep–Nov, spring) of enrollment, and other pathogens with significantly higher detection rates in cases compared to controls for the same specimen type. The adjusted odds ratios from these models were used to calculate the AF (AF = ((OR‐1)/OR) × 100) and to estimate the detection rate associated with illness (AF‐adjusted prevalence = observed detection rate × AF). The analysis was performed overall, stratified by admission diagnosis (respiratory vs. non‐respiratory) and infant HIV status.

Univariate and multivariable analyses to identify factors associated with hospitalisation among respiratory cases compared to controls were performed using a random effects multivariable logistic regression model accounting for site‐specific clustering. Variables that were significant at *p* < 0.2 on univariate analysis were evaluated in the multivariable model, and non‐significant factors (*p* > 0.05) dropped with stepwise backward selection. All 2‐way interactions were evaluated. Age group and HIV status were included in the model a priori. Sex, season of enrollment, underlying conditions, malnutrition (weight‐for‐age less than −2 standard deviations from WHO mean Z‐score), feeding type in the first 6 months of life, prematurity (gestational age < 37 weeks), low birth weight (< 2500 g), vaccination (up‐to‐date for age, using the *H. influenzae* type b vaccine given as part of the routine infant immunisation schedule at 6, 10, 14 weeks as a proxy), mother/caregiver highest education level, and pathogens for which the detection rate was significantly higher in cases compared to controls were evaluated in the model. Data on maternal characteristics such as maternal health, vital status, and age at delivery were not available. Statistical analyses were performed using Stata version 18.0.

## 3 Results

### 3.1 Infant Characteristics

**FIGURE 1:**
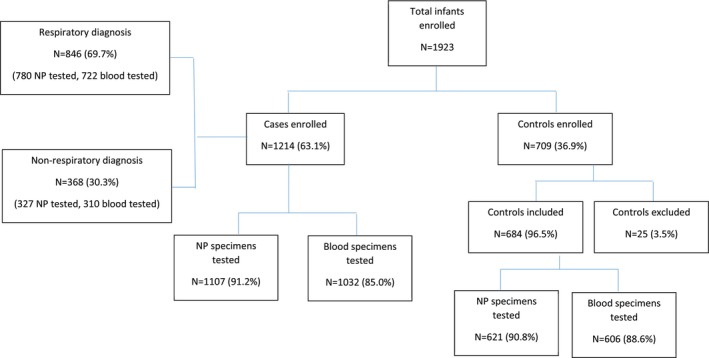
Flowchart of infants enrolled and specimens tested, by case status, South Africa, November 2016—October 2018. NP = nasopharyngeal aspirate.

Over the study period, 1923 infants were enrolled, 1214 (63.1%) cases and 709 (36.9%) controls (Figure 1). An average of 29, 35 and 15 controls, respiratory cases and non‐respiratory cases were enrolled per month, respectively (Supplementary Figure 1). At follow‐up, 3.5% (25/709) control infants reported symptoms within 14 days of enrollment and were excluded. Among hospitalised cases, 69.7% (846/1214) were admitted with respiratory illness and 30.3% (368/1214) with non‐respiratory illness. The majority of non‐respiratory cases were infants admitted with diarrhoea (193/368, 52.4%) or febrile seizures (45/368, 12.2%). Clinical characteristics of respiratory and non‐respiratory cases are described in Supplementary Table  1. Eight percent (68/846) and 1.7% (14/846) of respiratory cases were admitted to the intensive care unit and died in hospital, respectively, compared to 4.9% (18/368) and 2.7% (10/368) of non‐respiratory cases.

Compared to controls, a higher proportion of hospitalised infants had ≥ 1 underlying condition (2.6% vs. 0.4%), were malnourished (26.7% vs. 7.6%), born prematurely (18.3% vs. 9.2%), had a low birthweight (21.3% vs. 12.9%), had higher proportion of HIV exposure (40.7% vs. 25.9%) or were living with HIV (3.5% vs. 0.7%) (Table 1). Among respiratory cases 42.1% were aged < 3 months, 25.8% were 3–5 months and 32.2% were aged 6–11 months, whereas among non‐respiratory cases, 22.0% were aged < 3 months, 24.7% were 3–5 months and 53.3% were 6–11 months (Table 1 and Supplementary Figure 2).

**TABLE 1:** Characteristics of infants enrolled in the Infant Burden Study, South Africa, November 2016—October 2018 (*N* = 1898).

Characteristics	Controls, *n* (%)	All cases, *n* (%)	*p* value^f^	Respiratory cases, *n* (%)	Non‐respiratory cases, *n* (%)
	*N* = 684	*N* = 1214		*N* = 846	*N* = 368
Year					
2016–2017	352 (51.5)	539 (44.4)	**0.003**	368 (43.4)	172 (46.7)
2017–2018	332 (48.5)	675 (55.6)		479 (56.6)	196 (53.3)
Province					
Mpumalanga	139 (20.3)	214 (17.6)	**< 0.001**	157 (18.6)	57 (15.5)
KwaZulu‐Natal	218 (31.9)	641 (52.8)		430 (50.8)	211 (57.3)
North West	327 (47.8)	359 (29.6)		259 (30.6)	100 (27.2)
Season					
Summer	149 (21.8)	308 (25.4)	0.072	205 (24.2)	103 (28.0)
Autumn	183 (26.8)	326 (26.9)		248 (29.3)	78 (21.2)
Winter	162 (23.7)	303 (25.0)		202 (23.9)	101 (27.5)
Spring	190 (27.8)	277 (22.8)		191 (22.6)	86 (23.4)
Age group (months)					
0 to < 3	248 (36.3)	437 (36.0)	**0.002**	356 (42.1)	81 (22.0)
3 to < 6	220 (32.2)	309 (25.5)		218 (25.8)	91 (24.7)
6 to <12	216 (31.6)	468 (38.6)		272 (32.2)	196 (53.3)
Sex					
Male	334 (48.8)	698 (57.5)	**< 0.001**	489 (57.8)	209 (56.8)
Female	347 (50.7)	516 (42.5)		357 (42.2)	159 (43.2)
Unknown	3 (0.4)	0 (0.0)		0 (0.0)	0 (0.0)
Vaccinated for age^a^					
No	183 (26.8)	281 (23.2)	**0.036**	226 (26.7)	55 (15.0)
Yes	486 (71.1)	885 (72.9)		591 (69.9)	294 (79.9)
Unknown	15 (2.2)	48 (4.0)		29 (3.4)	19 (5.2)
Underlying condition^b^					
No	678 (99.1)	1182 (97.4)	**< 0.001**	821 (97.0)	361 (98.1)
Yes	3 (0.4)	32 (2.6)		25 (3.0)	7 (1.9)
Unknown	3 (0.4)	0 (0.0)		0 (0.0)	0 (0.0)
Malnutrition^c^					
No	624 (91.2)	889 (73.2)	**< 0.001**	621 (73.4)	268 (72.8)
Yes	52 (7.6)	324 (26.7)		224 (26.5)	100 (27.2)
Unknown	8 (1.2)	1 (0.1)		1 (0.1)	0 (0.0)
Premature birth^d^					
No	618 (90.4)	992 (81.7)	**< 0.001**	675 (79.8)	317 (86.1)
Yes	63 (9.2)	222 (18.3)		171 (20.2)	51 (13.9)
Unknown	3 (0.4)	0 (0.0)		0 (0.0)	0 (0.0)
Birthweight^e^					
Normal	561 (82.0)	891 (73.4)	**< 0.001**	609 (72.0)	282 (76.6)
Low	88 (12.9)	259 (21.3)		196 (23.2)	63 (17.1)
Unknown	35 (5.1)	64 (5.3)		41 (4.9)	23 (6.3)
HIV status					
HUU	352 (51.5)	652 (53.7)	**< 0.001**	482 (57.0)	170 (46.2)
HEU	177 (25.9)	494 (40.7)		313 (37.0)	181 (49.2)
ILWH	5 (0.7)	42 (3.5)		34 (4.0)	8 (2.2)
Unknown	150 (21.9)	26 (2.1)		17 (2.0)	9 (2.5)
Feeding type					
Exclusive breastfeeding	446 (65.2)	648 (53.4)	**< 0.001**	480 (56.7)	168 (45.7)
Mixed feeding	123 (18.0)	221 (18.2)		148 (17.5)	73 (19.8)
Formula feeding	99 (14.5)	305 (25.1)		195 (23.1)	110 (29.9)
Unknown	16 (2.3)	40 (3.3)		23 (2.7)	17 (4.6)
Caregiver education level					
Primary	356 (52.1)	554 (45.6)	**0.021**	394 (46.6)	160 (43.5)
Secondary	284 (41.5)	581 (47.9)		390 (46.1)	191 (51.9)
Tertiary	34 (5.0)	69 (5.7)		54 (6.4)	15 (4.1)
Unknown	10 (1.5)	10 (0.8)		8 (1.0)	2 (0.5)

Abbreviations: HEU, HIV exposed uninfected; HUU, HIV unexposed uninfected; ILWH, infants living with HIV.

^a^ Vaccination defined as full vaccine coverage for age using the hexavalent DTaP‐IPV‐Hib‐HBV (diphtheria, tetanus, acellular pertussis, inactivated polio vaccine, *Haemophilus Influenzae* type B and hepatitis B) vaccine given as part of the routine infant immunisation schedule at 6, 10 and 14 weeks of age with a booster at 18 months.

^b^ Underlying condition includes asthma, chronic lung, heart, liver or renal disease, stroke, organ transplant, anaemia, immunosuppressive therapy, splenectomy, burns, immunoglobulin deficiency, autoimmune disease, nephrotic syndrome, cancer, spinal cord injury, seizure disorder, cerebral palsy, congenital heart disease, other congenital disorder, obesity or chronic gastrointestinal problems.

^c^ Malnutrition defined as a weight‐for‐age less than −2 standard deviations from the WHO mean Z‐score.

^d^ Premature defined as gestational age at birth of < 37 weeks.

^e^ Low infant birthweight defined as < 2500 g.

^f^ Chi‐squared test.

### 3.2 Pathogen Detection Rate

In nasopharyngeal specimens collected from respiratory cases, *S. pneumoniae* (39.9%, 311/780), *H. influenzae* (35.5%, 277/780), *M. catarrhalis* (34.1%, 266/780), rhinovirus (32.3%, 252/780) and RSV (29.4%, 229/780) were most commonly detected (Supplementary Table 2). Among non‐respiratory cases, *S. pneumoniae* (45.6%, 149/327), *M. catarrhalis* (41.6%, 136/327) and rhinovirus (32.7%, 107/327) were most prevalent. In nasopharyngeal specimens, comparing all hospitalised cases to control infants, RSV (22.2% vs. 3.2%, *p* < 0.001) and *K. pneumoniae* (13.7% vs. 8.5%, *p* = 0.001) had a significantly higher detection rate (Supplementary Table 2). When comparing respiratory cases to controls, influenza (4.2% vs. 1.3%, *p* = 0.001), RSV (29.4% vs. 3.2%, *p* < 0.001) and HMPV (5.5% vs. 2.1%, *p* = 0.001) had a significantly higher detection rate. Among non‐respiratory cases compared to controls, only *K. pneumoniae* was detected at a significantly higher prevalence (15.3% vs. 8.5%, *p* = 0.001).

In blood specimens collected from respiratory cases, cytomegalovirus (31.4%, 227/722), *E. coli* (9.3%, 67/722) and *K. pneumoniae* (5.1%, 37/722) had the highest detection rates, whereas in non‐respiratory cases, cytomegalovirus (28.1%, 87/310) and *E. coli* (12.3%, 38/310) were also most prevalent. When comparing the detection rate of pathogens in blood specimens among all cases, respiratory cases and non‐respiratory cases to controls, no pathogens were found to have a significant difference in detection rate (Supplementary Table  2).

### 3.3 Pathogen Aetiological Fraction

AF was determined for the pathogens that were identified at a higher detection rate in hospitalised infants compared to control infants in nasopharyngeal specimens. Among respiratory cases, RSV (94.0%, 95%CI 89.2–96.7%), influenza (72.6%, 95%CI 35.1–88.4%), HMPV (74.9%, 95%CI 47.9–87.9%) and *K. pneumoniae* (48.0%, 95%CI 18.7–66.8%) had a significant AF (Table 2). Among non‐respiratory cases, only *K. pneumoniae* (60.7%, 95%CI 34.7–76.3%) had a significant AF.

**TABLE 2:** Aetiological fraction and AF‐adjusted prevalence of pathogens^a^ stratified by admission diagnosis among infants aged < 1 year enrolled in the Infant Burden Study, South Africa, November 2016—October 2018.

Pathogen^a^	All cases			Respiratory cases			Non‐respiratory cases		
	Observed detection rate, *n*/*N* (%)	AF^b^ % (95%CI)	AF‐adjusted prevalence %	Observed detection rate, *n*/*N* (%)	AF^b^ % (95%CI)	AF‐adjusted prevalence %	Observed detection rate, *n*/*N* (%)	AF^b^ % (95%CI)	AF‐adjusted prevalence %
RSV	246/1107 (22.2)	**91.4 (84.6; 95.2)**	20.3	229/780 (29.4)	**94.0 (89.2; 96.7)**	27.6	17/327 (5.2)	49.5 (−21.7; 79.1)	2.6
Influenza^c^	39/1107 (3.5)	**66.9 (24.1; 85.5)**	2.3	33/780 (4.2)	**72.6 (35.1; 88.4)**	3.0	6/327 (1.8)	44.8 (−77.1; 82.8)	0.8
Human metapneumovirus	51/1107 (4.6)	**66.2 (31.7; 83.3)**	3.0	43/780 (5.5)	**74.9 (47.9; 87.9)**	4.1	8/327 (2.5)	25.4 (−100.8; 72.3)	0.6
*Klebsiella pneumoniae*	152/1107 (13.7)	**53.7 (30.4; 69.2)**	7.4	102/780 (13.1)	**48.0 (18.7; 66.8)**	6.3	50/327 (15.3)	**60.7 (34.7; 76.3)**	9.3

Abbreviation: AF, aetiological fraction.

^a^ Pathogens were included if they had a significantly higher detection rate in cases (all, respiratory or non‐respiratory) compared to controls (RSV, influenza, HMPV and *Klebsiella pneumoniae* in nasopharyngeal aspirates).

^b^ Obtained from models adjusted for age, HIV exposure/infection status, season and other pathogens that had significantly higher detection rate in cases compared to controls in the same specimen type—RSV, influenza, HMPV and *K. pneumoniae* in nasopharygeal aspirates. No pathogens were identified with significantly different detection rates in blood specimens.

^c^ Includes influenza A and influenza B viruses.

Among HUU infants, RSV (95.0%, 95%CI 89.0–97.8%), HMPV (74.4%, 95%CI 39.2–89.2%) and *K. pneumoniae* (57.5%, 95%CI 27.3–75.2%) were significantly attributable to illness (Table 3). In HEU infants, only RSV (74.0%, 37.6–89.1%) was found to have a significant AF, and in ILWH, no pathogens were found to have a significant AF, although likely due to small numbers.

**TABLE 3:** Aetiological fraction and AF‐adjusted prevalence of pathogens^a^ stratified by HIV status among infants aged < 1 year enrolled in the Infant Burden Study, South Africa, November 2016—October 2018.

Pathogen^a^	HUU			HEU			ILWH		
	Observed detection rate, *n*/*N* (%)	AF^b^ % (95%CI)	AF‐adjusted prevalence %	Observed detection rate, *n*/*N* (%)	AF^b^ % (95%CI)	AF‐adjusted prevalence %	Observed detection rate, *n*/*N* (%)	AF^b^ % (95%CI)	AF‐adjusted prevalence %
RSV	181/645 (28.0)	**95.0 (89.0; 97.8)**	26.6	72/491 (14.7)	**74.0 (37.6; 89.1)**	10.9	3/41 (7.1)	−68.1 (−4632.7; 94.0)	Not determined
Influenza^c^	13/645 (2.0)	66.8 (−9.7; 90.0)	1.3	23/491 (4.7)	67.7 (−3.4; 89.9)	3.2	1/42 (2.4)	−101.1 (−8993.4; 95.6)	Not determined
Human metapneumovirus	37/645 (5.7)	**74.4 (39.2; 89.2)**	4.2	15/491 (3.1)	27.4 (−121.1; 76.2)	0.8	1/42 (2.4)	−952.9 (−112270.9; 90.1)	Not determined
*Klebsiella pneumoniae*	76/623 (12.2)	**57.5 (27.3; 75.2)**	7.0	69/472 (14.6)	45.5 (−1.6; 70.8)	6.6	7/42 (16.7)	73.0 (−665.7; 99.0)	12.2

Abbreviations: AF, aetiological fraction; HEU, HIV exposed uninfected; HUU, HIV unexposed uninfected; ILWH, infants living with HIV.

^a^ Pathogens were included if they had a significantly higher detection rate in cases (all, respiratory or non‐respiratory) compared to controls (RSV, influenza, HMPV and *Klebsiella pneumoniae* in nasopharyngeal aspirates).

^b^ Obtained from models adjusted for age, HIV exposure/infection status, season and other pathogens that had significantly higher detection rate in cases compared to controls in the same specimen type—RSV, influenza, HMPV and *K. pneumoniae* in nasopharygeal aspirates. No pathogens were identified with significantly different detection rates in blood specimens.

^c^ Includes influenza A and influenza B viruses.

### 3.4 Factors Associated With Respiratory Illness Hospitalisation

**FIGURE 2:**
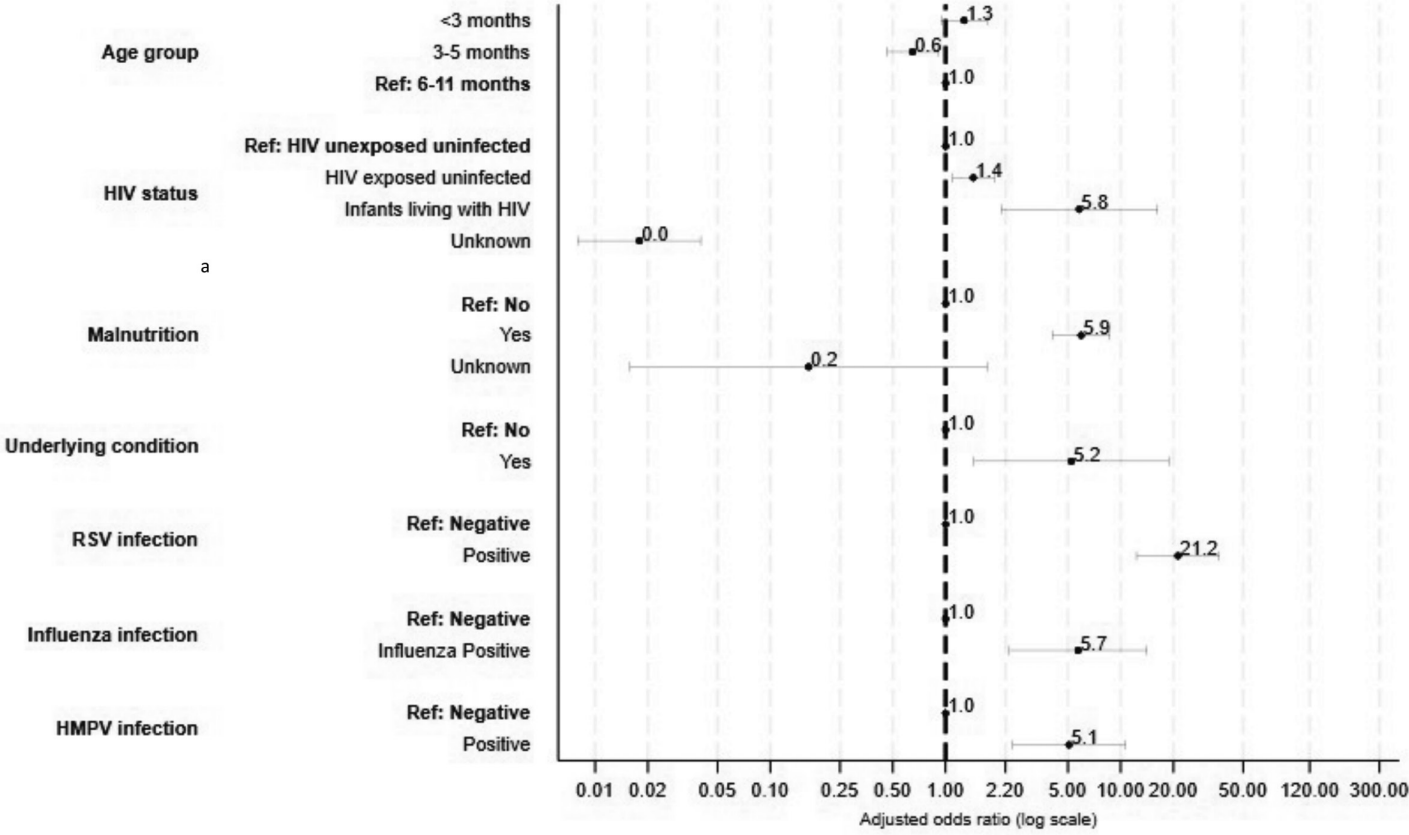
Factors associated with hospitalisation among infants aged < 1 year with a respiratory admission diagnosis^c^, South Africa, November 2016—October 2018. ^a^Malnutrition defined as a weight‐for‐age less than −2 standard deviations from the WHO mean Z‐score. ^b^Underlying condition includes any of the following: asthma, chronic lung, heart, liver or renal disease, stroke, sinusitis, organ transplant, anaemia, immunosuppressive therapy, splenectomy, diabetes, burns immunoglobulin deficiency, autoimmune disease, nephrotic syndrome, cancer, spinal cord injury, seizure disorder, cerebral palsy, congenital heart disease, other congenital disorder, obesity or chronic gastrointestinal problems. ^c^Mixed effects regression model, accounting for clustering by site.

On multivariable analysis, infants were more likely to be admitted to hospital with a respiratory illness if they were HEU (aOR 1.5, 95%CI 1.1–2.0) or ILWH (aOR 6.6, 95%CI 2.1–20.5) compared to HUU, had ≥ 1 underlying condition (aOR 4.8, 95%CI 1.3–17.6), were malnourished (aOR 6.0, 95%CI 4.0–8.9) or were infected with RSV (aOR 19.7, 95%CI 11.4–34.1), influenza (aOR 5.7, 95%CI 2.3–14.1) or HMPV (aOR 4.1, 95%CI 2.0–8.6) (Figure 2 and Supplementary Table 3).

### 3.5 Serological Influenza Attack Rate

Paired sera was obtained for 54.1% (370/684), 44.7% (378/846) and 44.3% (163/368) of controls, respiratory cases and non‐respiratory cases, respectively. The median time between enrollment and follow‐up serum collection was 23 days (interquartile range [IQR] 21–28) in controls, 22 days (IQR 21–27 days) in respiratory cases and 22 days (IQR 20–27 days) in non‐respiratory cases. For any influenza subtypes/lineages, the proportion of infants with HAI GMTs ≥ 1:40 at enrollment were higher for respiratory cases than controls (Supplementary Table 5). The serological influenza attack rate was 13.8% (51/370) in controls, 17.2% (65/378) in respiratory cases and 11.7% (19/163) in non‐respiratory cases. The serological influenza attack rate did not differ among respiratory cases compared to controls for all influenza subtypes/lineages (Supplementary Table 4). Similarly, when comparing the serological attack rate between respiratory cases and controls separately for the year in which a specific subtype/lineage was predominant (A(H3N2) in 2017 and A(H1N1)pdm09 in 2018), no significant differences were observed.

## 4 Discussion

Further progress towards reducing the remaining high burden of respiratory illness and mortality in infants will rely on pathogen‐directed interventions, and prioritisation of resources, especially in low‐ and middle‐income countries. We found RSV to be the leading cause of respiratory illness hospitalisation in infants, and infection with RSV, influenza or HMPV increased the risk of respiratory illness hospitalisation among infants. In addition, non‐pathogen‐related factors including HIV exposure and living with HIV, having an underlying condition and being malnourished increased an infant's risk of hospitalisation for respiratory illness.

In our study, RSV was found to be the most important pathogen causing respiratory illness hospitalisation in infants, with an AF of 94% in respiratory cases. RSV infection also increased the risk of respiratory illness hospitalisation by almost 20‐fold. This finding re‐affirms findings from other studies in young children [4, 11]. The PERCH study, conducted in 2011–2014 at nine study sites globally among children aged 1–59 months hospitalised with pneumonia, found RSV to have the largest AF (31%, 95% credible interval 28.4–34.2) [3]. At the South African study site, RSV was the leading pathogen among HUU (aetiologic fraction 36.4%, 95% credible interval 30.5–43.1) and HEU (aetiologic fraction 31.6%, 95% credible interval 24.8–38.8) children [12]. In the PERCH study, RSV was also found to be the 4th leading pathogen among children living with HIV (9.7%, credible interval 2.2–14.6), after *Pneumocystis jirovecii, S. aureus* and *S. pneumoniae* [13]*,* whereas in our study, we were not able to determine aetiological fraction in this group due to low numbers.

After RSV, influenza and HMPV were found to have the highest AF (73% and 75%, respectively) and to be associated with respiratory illness hospitalisation. The influenza AF in infants in our study was lower than that previously described in the surveillance programme in 2012–2016 (93.5% in infants with severe acute respiratory illness and 87.6% in infants with severe chronic respiratory illness) [14]. However, our study only included 2 years, was conducted after the previous study and included an additional sentinel site in Mpumalanga Province. Both studies, however, support the importance of influenza as a cause of severe respiratory illness in infants. In a systematic analysis of the global burden of influenza in paediatric respiratory hospitalisations, among infants aged < 1 year, the median percentage of influenza positives was 4% (IQR 2–7) [15], similar to the detection rate in our study. In our setting, HMPV was also found to be an important contributor to infant respiratory illness hospitalisations. A case–control study in four countries (Albania, Jordan, Nicaragua and the Philippines) found HMPV to be a leading cause of LRTI hospitalisations in infants [16]. Studies in South Africa in 2009–2013 [17] as well as a global systematic review of studies between 2001 and 2019 [18] found that infants have the highest burden of HMPV‐associated illness.

*Klebsiella pneumoniae* was found to be a significant cause of hospitalisation in infants hospitalised with respiratory illness and non‐respiratory illness. *K. pneumoniae* is a common cause of neonatal sepsis and nosocomial pneumonia [19], and in the CHAMPS study was responsible for 30% of deaths in infants aged 1–11 months [20]. However, the role of *K. pneumoniae* in community‐acquired pneumonia is less well understood. In a case–control study in South Africa among infants with LRTI in 2012–2015, *K. pneumoniae* was detected in nasopharyngeal specimens in 16% of LRTI episodes among infants with a median age of 3.7 months, and was significantly associated with LRTI (OR 1.93, 95% CI 1.25–3.03) [21].

Other factors associated with an increased risk of respiratory illness hospitalisation included HIV exposure and infection, having underlying illness and being malnourished. While HIV infection is well known to increase the risk of severe disease and mortality [22], in our setting of a mature programme for the prevention of vertical HIV transmission, there is a growing population of HEU infants at risk of more severe disease [9]. In our study, malnourished infants had a 6‐fold higher risk of respiratory illness hospitalisation compared to well‐nourished children. Studies on the causes of infant and childhood respiratory illness and death have consistently shown malnutrition to be a leading risk factor [23], with malnutrition identified as a causal factor in 40% of deaths in children aged < 5 years in the CHAMPS study [24]. Measures to improve socioeconomic factors, nutrition and to reduce HIV‐infection and HIV‐exposure will also be important to reduce the high burden of severe respiratory illness in infants.

While the influenza detection rate using serology was higher among respiratory cases (17.2%) than control infants (13.8%), it did not differ significantly. The influenza detection rate in control infants was 1.3% using PCR and 13.8% using serology, and among respiratory cases was 4.2% by PCR and 17.2% using serology. Although serology detected a higher number of influenza infections compared to PCR, it was not useful in assigning the attribution of illness due to influenza infection. The high serological detection rate in control infants may be due to asymptomatic influenza infections. A community cohort study in South Africa found that 44% of influenza infections among individuals of all ages, and 21% of infants aged < 1 year were asymptomatic [25]. The higher enrollment titres in cases compared to controls also indicated that at the time of study enrollment, hospitalised infants were already mounting an immune response to the influenza infection, which would have reduced the ability to detect a four‐fold rise in titres between paired sera.

The strengths of our study include the enrollment of asymptomatic control infants which enabled us to attribute aetiology, as well as the inclusion of hospitalised infants with non‐respiratory symptoms to better understand aetiology in infants that do not present with typical respiratory symptoms. Our study also had a number of limitations. Firstly, our study took place over a period of 2 years, which was subject to pathogen circulation during this time period. Secondly, we were underpowered to examine aetiology by HIV status and were therefore only able to report results overall in infants. We did not detect pathogens in blood specimens that were significantly attributable to illness, which was likely due to the small sample size and the rarity of pathogen detection in blood. Thirdly, we did not collect lower respiratory tract samples, such as bronchoalveolar lavage, and we may therefore have underestimated the contribution of bacteria to infant hospitalisation. However, our methodology and results were similar to a number of other infant aetiology studies [12, 16]. Fourthly, our study was conducted in a middle‐income setting, with high coverage of childhood vaccines such as the pneumococcal conjugate vaccine and *H. influenzae* type b vaccine, and may therefore not be generalisable to settings where childhood vaccination coverage is lower. Fifthly, our study was conducted prior to the emergence of SARS‐CoV‐2, and therefore, we were not able to determine the contribution of SARS‐CoV‐2 to infant hospitalisations. While studies have shown SARS‐CoV‐2 to be an important cause of infant hospitalisation in our setting [26], further studies are needed to better understand the aetiological fraction in the post‐pandemic period.

There were also some limitations to our study design. Controls were infants presenting for immunisation and may have differed from the cases in terms of healthcare seeking behaviour; however, the proportion of infants vaccinated according to their age was similar in both groups (27% of controls and 24% of cases). Due to the case control study design, we used odds ratios instead of risk ratios for the calculation of aetiological fractions. The odds ratio provides a reasonable approximation of the risk ratio when the outcome is rare. In South Africa, RSV has the highest rate of medically attended severe respiratory illness in infants at 3588 per 100,000 population [27], thereby satisfying the rare disease assumption. While we adjusted for geographical seasons, we did not conduct virus‐specific seasonal analysis due to the multiple pathogens included in our study; however, surveillance data show that the RSV and influenza seasons in South Africa typically occur in autumn and winter, respectively [22, 28]. The follow up visit was conducted between 2 and 6 weeks post‐enrollment; this range in follow up period may have affected the serological attack rates, with infants with a longer follow up time having had more time to become infected and develop an immune response.

In South Africa, as well as in other resource‐limited settings with a disproportionate burden of infant morbidity and deaths, understanding the causes of illness is important to target interventions and guide policy. The introduction of interventions for preventing severe RSV disease, such as maternal RSV immunisation [6] and long‐acting monoclonal antibodies [29] should be prioritised. In addition, influenza vaccines administered to pregnant women to prevent influenza‐associated hospitalisation in young infants [30] should be encouraged.

## Author Contributions

All authors approved the final manuscript as submitted and agree to be accountable for all aspects of the work. Conceptualisation—CC, SW, AvG, ST, JM. Data curation—SW, M Makhasi, CR, NW. Formal analysis—NW. Funding acquisition—CC, SW. Investigation and methodology—NW, SW, AvG, ST, JM, CvM, FT, OH KS, M Moleleki, CR, LM, NB, CC. Writing (original draft preparation)—NW. Writing (review and editing)—NW, SW, AvG, ST, JM, CvM, FT, OH, KS, M Moleleki, CR, LM, MM, NB, CC.

## Ethics Statement

Ethical approval was obtained from the University of the Witwatersrand Human Research Ethics Committee (M140824) and University of KwaZulu‐Natal Biomedical Research Ethics Committee (BE605/16). This surveillance was deemed non‐research by the US Centers for Disease Control and Prevention.

## Consent

Written informed consent for study participation was obtained from parents/caregivers of study participants by trained surveillance officers on interview prior to any study procedures being performed.

## Conflicts of Interest

CC has received grant support from Sanofi, the Gates Foundation, US CDC, South African Medical Research Council and Wellcome Trust. AvG and NW have received grant funding from the US CDC, the Gates Foundation and Sanofi. JM has received grant funding from Sanofi. CVM has received grant funding from Pfizer. SW received grant funding from the US CDC and the Gates Foundation. All other co‐authors have no conflicts of interest.

## Peer Review

The peer review history for this article is available at https://www.webofscience.com/api/gateway/wos/peer‐review/10.1111/irv.70135.

## Disclaimer

The findings and conclusions in this paper are those of the authors and do not necessarily represent the views of their affiliated institutions or the agencies funding the study.

## Supporting information


**Table S1** Clinical characteristics of hospitalised infants enrolled in the Infant Burden Study, South Africa, November 2016—October 2018 (*N* = 1214).
**Table S2** Pathogen‐specific prevalence in cases and controls among infants aged < 1 year, South Africa, November 2016—October 2018.
**Table S3** Factors associated with hospitalisation among infants aged < 1 year with a respiratory admission diagnosis, South Africa, November 2016—October 2018.
**Table S4** Serological influenza attack rate^a^ among infants aged < 1 year, South Africa, November 2016—October 2018.
**Table S5**Proportion of infants aged > 1 year with haemagglutination inhibition (HAI) titres (≥ 1:40) at enrollment, South Africa, November 2016—October 2018.
**Figure S1** Number of infants enrolled in the Infant Burden Study by year and month, South Africa, November 2016—October 2018 (*N* = 1923).
**Figure S2** Percentage of infants enrolled in the Infant Burden Study by study year, case definition and age group, South Africa, November 2016—October 2018 (*N* = 1923).

## Data Availability

The data generated and analysed during this study contain potentially identifiable information and therefore have restricted access due to privacy and ethical issues. Access to aggregated data can be obtained by request to the corresponding author, Nicole Wolter (nicolew@nicd.ac.za), and will be subject to proof of an IRB‐approved protocol and signature of a data sharing agreement. Responses to requests will be within 3 weeks from request receipt.

## References

[1] United Nations Inter‐agency Group for Child Mortality Estimation , “Levels and Trends in Childhood Mortality: Report 2024,” accessed May 13, 2025, https://data.unicef.org/resources/levels‐and‐trends‐in‐child‐mortality‐2024/?utm_campaign=IGME%202025&utm_medium=email&utm_source=Mailjet.

[2] S. Mahtab , D. M. Blau , Z. J. Madewell , et al., “Post‐Mortem Investigation of Deaths due to Pneumonia in Children Aged 1‐59 Months in Sub‐Saharan Africa and South Asia From 2016 to 2022: An Observational Study,” Lancet Child and Adolescent Health 8 (2024): 201–213. 38281495 10.1016/S2352-4642(23)00328-0 PMC10864189

[3] K. L. O'Brien , H. C. Baggett , A. Greenbaum , et al., “Causes of Severe Pneumonia Requiring Hospital Admission in Children Without HIV Infection From Africa and Asia: The PERCH Multi‐Country Case‐Control Study,” Lancet 394 (2019): 757–779. 31257127 10.1016/S0140-6736(19)30721-4 PMC6727070

[4] S. Jain , D. J. Williams , S. R. Arnold , et al., “Community‐Acquired Pneumonia Requiring Hospitalization Among U.S. Children,” New England Journal of Medicine 372 (2015): 835–845. 25714161 10.1056/NEJMoa1405870 PMC4697461

[5] H. L. Moline , A. Tannis , A. P. Toepfer , et al., “Early Estimate of Nirsevimab Effectiveness for Prevention of Respiratory Syncytial Virus‐Associated Hospitalization Among Infants Entering Their First Respiratory Syncytial Virus Season ‐ New Vaccine Surveillance Network, October 2023‐February 2024,” Morbidity and Mortality Weekly Report 73 (2024): 209–214. 38457312 10.15585/mmwr.mm7309a4 PMC10932582

[6] B. Kampmann , S. A. Madhi , I. Munjal , et al., “Bivalent Prefusion F Vaccine in Pregnancy to Prevent RSV Illness in Infants,” New England Journal of Medicine 388 (2023): 1451–1464. 37018474 10.1056/NEJMoa2216480

[7] J. Moyes , S. Tempia , S. Walaza , et al., “The Burden of RSV‐Associated Illness in Children Aged < 5 Years, South Africa, 2011 to 2016,” BMC Medicine 21 (2023): 139. 37038125 10.1186/s12916-023-02853-3 PMC10088270

[8] M. F. Flynn , M. Kelly , and J. S. G. Dooley , “Nasopharyngeal Swabs vs. Nasal Aspirates for Respiratory Virus Detection: A Systematic Review,” Pathogens 10, no. 11 (2021): 1515. 34832670 10.3390/pathogens10111515 PMC8620365

[9] C. Cohen , J. Moyes , S. Tempia , et al., “Epidemiology of Acute Lower Respiratory Tract Infection in HIV‐Exposed Uninfected Infants,” Pediatrics 137 (2016): e20153272. 27025960 10.1542/peds.2015-3272 PMC9075335

[10] World Health Organization , “WHO Global Influenza Surveillance Network: Manual for the Laboratory Diagnosis and Virological Surveillance of Influenza,” (2011).

[11] J. Kubale , S. Kujawski , I. Chen , et al., “Etiology of Acute Lower Respiratory Illness Hospitalizations Among Infants in 4 Countries,” Open Forum Infectious Diseases 10 (2023): ofad580. 38130597 10.1093/ofid/ofad580 PMC10733183

[12] D. P. Moore , V. L. Baillie , A. Mudau , et al., “The Etiology of Pneumonia in HIV‐Uninfected South African Children: Findings From the Pneumonia Etiology Research for Child Health (PERCH) Study,” Pediatric Infectious Disease Journal 40 (2021): S59–S68. 34448745 10.1097/INF.0000000000002650 PMC8448398

[13] D. P. Moore , V. L. Baillie , A. Mudau , et al., “The Etiology of Pneumonia in HIV‐1‐Infected South African Children in the Era of Antiretroviral Treatment: Findings From the Pneumonia Etiology Research for Child Health (PERCH) Study,” Pediatric Infectious Disease Journal 40 (2021): S69–S78. 34448746 10.1097/INF.0000000000002651 PMC8448402

[14] S. Tempia , S. Walaza , J. Moyes , et al., “Attributable Fraction of Influenza Virus Detection to Mild and Severe Respiratory Illnesses in HIV‐Infected and HIV‐Uninfected Patients, South Africa, 2012–2016,” Emerging Infectious Diseases 23 (2017): 1124–1132. 28628462 10.3201/eid2307.161959 PMC5512492

[15] K. E. Lafond , H. Nair , M. H. Rasooly , et al., “Global Role and Burden of Influenza in Pediatric Respiratory Hospitalizations, 1982–2012: A Systematic Analysis,” PLoS med 13 (2016): e1001977. 27011229 10.1371/journal.pmed.1001977 PMC4807087

[16] J. Kubale , S. Kujawski , I. Chen , et al., “Etiology of Acute Lower Respiratory Illness Hospitalizations Among Infants in 4 Countries,” Open Forum Infectious Diseases 10, no. 12 (2023): ofad580. 38130597 10.1093/ofid/ofad580 PMC10733183

[17] M. J. Groome , J. Moyes , C. Cohen , et al., “Human Metapneumovirus‐Associated Severe Acute Respiratory Illness Hospitalisation in HIV‐Infected and HIV‐Uninfected South African Children and Adults,” Journal of Clinical Virology 69 (2015): 125–132. 26209394 10.1016/j.jcv.2015.06.089 PMC9134797

[18] X. Wang , Y. Li , M. Deloria‐Knoll , et al., “Global Burden of Acute Lower Respiratory Infection Associated With Human Metapneumovirus in Children Under 5 Years in 2018: A Systematic Review and Modelling Study,” Lancet Glob Health 9 (2021): e33–e43. 33248481 10.1016/S2214-109X(20)30393-4 PMC7783516

[19] R. C. Mashau , S. T. Meiring , A. Dramowski , et al., “Culture‐Confirmed Neonatal Bloodstream Infections and Meningitis in South Africa, 2014‐19: A Cross‐Sectional Study,” Lancet Global Health 10, no. 8 (2022): e1170–e1178. 35839815 10.1016/S2214-109X(22)00246-7 PMC9296659

[20] J. R. Verani , D. M. Blau , E. S. Gurley , et al., “Child Deaths Caused by *Klebsiella pneumoniae* in Sub‐Saharan Africa and South Asia: A Secondary Analysis of Child Health and Mortality Prevention Surveillance (CHAMPS) Data,” Lancet Microbe 5 (2024): e131–e141. 38218193 10.1016/S2666-5247(23)00290-2 PMC10849973

[21] H. J. Zar , R. MacGinty , L. Workman , et al., “ *Klebsiella pneumoniae* Lower Respiratory Tract Infection in a South African Birth Cohort: A Longitudinal Study,” International Journal of Infectious Diseases 121 (2022): 31–38. 35472523 10.1016/j.ijid.2022.04.043 PMC9174060

[22] C. Cohen , S. Walaza , J. Moyes , et al., “Epidemiology of Viral‐Associated Acute Lower Respiratory Tract Infection Among Children <5 Years of Age in a High HIV Prevalence Setting, South Africa, 2009‐2012,” Pediatric Infectious Disease Journal 34 (2015): 66–72. 25093972 10.1097/INF.0000000000000478 PMC4276570

[23] C. Troeger , B. Blacker , I. A. Khalil , et al., “Estimates of the Global, Regional, and National Morbidity, Mortality, and Aetiologies of Lower Respiratory Infections in 195 Countries, 1990–2016: A Systematic Analysis for the Global Burden of Disease Study 2016,” Lancet Infect dis 18 (2018): 1191–1210. 30243584 10.1016/S1473-3099(18)30310-4 PMC6202443

[24] Z. J. Madewell , A. M. Keita , P. M.‐G. Das , et al., “Contribution of Malnutrition to Infant and Child Deaths in Sub‐Saharan Africa and South Asia,” BMJ Global Health 9 (2024): e017262. 10.1136/bmjgh-2024-017262 PMC11624724 39638608

[25] C Cohen , J Kleynhans , J Moyes , et al., “Asymptomatic Transmission and High Community Burden of Seasonal Influenza in an Urban and a Rural Community in South Africa, 2017–18 (PHIRST):” A Population Cohort Study, (2021): 863 p. 10.1016/S2214-109X(21)00141-8 PMC8262603 34019838

[26] N. Chiwandire , W. Jassat , M. Groome , et al., “Changing Epidemiology of COVID‐19 in Children and Adolescents Over Four Successive Epidemic Waves in South Africa, 2020‐2022,” Journal of the Pediatric Infectious Diseases Society 12 (2023): 128–134. 36648247 10.1093/jpids/piad002 PMC10112681

[27] S. Tempia , J. Moyes , A. L. Cohen , et al., “The National Burden of Influenza‐Like Illness and Severe Respiratory Illness Overall and Associated With Nine Respiratory Viruses in South Africa, 2013‐2015,” Influenza Other Respir Viruses 16 (2022): 438–451. 35150059 10.1111/irv.12949 PMC8983907

[28] J. M. McAnerney , C. Cohen , J. Moyes , et al., “Twenty‐Five Years of Outpatient Influenza Surveillance in South Africa, 1984‐2008,” Journal of Infectious Diseases 206 (2012): 153–158. 10.1093/infdis/jis575 23169963

[29] L. L. Hammitt , R. Dagan , Y. Yuan , et al., “Nirsevimab for Prevention of RSV in Healthy Late‐Preterm and Term Infants,” New England Journal of Medicine 386 (2022): 837–846. 35235726 10.1056/NEJMoa2110275

[30] M. C. Nunes , S. Walaza , S. Meiring , et al., “Effectiveness of Influenza Vaccination of Pregnant Women for Prevention of Maternal and Early Infant Influenza‐Associated Hospitalizations in South Africa: A Prospective Test‐Negative Study,” Open Forum Infectious Diseases 9, no. 11 (2022): ofac552. 36447608 10.1093/ofid/ofac552 PMC9697604

