# Supplementary material for: Aetiological Fraction of Influenza, Respiratory Syncytial Virus and Other Respiratory Pathogens in Infants Aged < 1 Year Hospitalised With Respiratory and Non‐Respiratory Medical Illness in South Africa, 2016–2018
Source: Influenza Other Respir Viruses. 2025 Jul 1;19(7):e70135. doi: 10.1111/irv.70135 (PMC12210142; doi:10.1111/irv.70135)
Supplement: Supplementary file 1 — Table S1 Clinical characteristics of hospitalised infants enrolled in the Infant Burden Study, South Africa, November 2016—October 2018 (N = 1214). Table S2 Pathogen‐specific prevalence in cases and controls among infants aged < 1 year, South Africa, November 2016—October 2018. Table S3 Factors associated with hospitalisation among infants aged < 1 year with a respiratory admission diagnosis, South Africa, November 2016—October 2018. Table S4 Serological influenza attack ratea among infants aged < 1 year, South Africa, November 2016—October 2018. Table S5Proportion of infants aged > 1 year with haemagglutination inhibition (HAI) titres (≥ 1:40) at enrollment, South Africa, November 2016—October 2018. Figure S1 Number of infants enrolled in the Infant Burden Study by year and month, South Africa, November 2016—October 2018 (N = 1923). Figure S2 Percentage of infants enrolled in the Infant Burden Study by study year, case definition and age group, South Africa, November 2016—October 2018 (N = 1923). [file IRV-19-e70135-s001.docx]

**Supplementary table 1.** Clinical characteristics of hospitalised infants enrolled in the Infant Burden Study, South Africa, November 2016 – October 2018 (N=1214)

| Characteristic | All cases  n (%)  N=1214 | Respiratory cases^a^  n (%)  N=846 | Non-respiratory cases^b^  n (%)  N=368 |
| --- | --- | --- | --- |
| Symptom duration (days) |  |  |  |
| ≤2 | 776 (63.9) | 516 (61.0) | 260 (70.7) |
| 3-7 | 339 (27.9) | 258 (30.5) | 81 (22.0) |
| ≥8 | 75 (6.2) | 60 (7.1) | 15 (4.1) |
| Unknown | 24 (2.0) | 12 (1.4) | 12 (3.3) |
| Cough |  |  |  |
| No | 403 (33.2) | 128 (15.1) | 275 (74.7) |
| Yes | 811 (66.8) | 718 (84.9) | 93 (25.3) |
| Fever |  |  |  |
| No | 435 (35.8) | 264 (31.2) | 171 (46.5) |
| Yes | 779 (64.2) | 582 (68.8) | 197 (53.5) |
| Supplemental oxygen |  |  |  |
| No | 763 (62.9) | 434 (51.3) | 329 (89.4) |
| Yes | 451 (37.2) | 412 (48.7) | 39 (10.6) |
| ICU admission |  |  |  |
| No | 1128 (92.9) | 778 (92.0) | 350 (95.1) |
| Yes | 86 (7.1) | 68 (8.0) | 18 (4.9) |
| Antibiotics administered |  |  |  |
| No | 114 (9.4) | 31 (3.7) | 83 (22.6) |
| Yes | 1100 (90.6) | 815 (96.3) | 285 (77.5) |
| Hospital duration (days) |  |  |  |
| <2 | 120 (9.9) | 70 (8.3) | 50 (13.6) |
| 2-4 | 545 (44.9) | 360 (42.6) | 185 (50.3) |
| 5-7 | 264 (21.8) | 203 (24.0) | 61 (16.6) |
| ≥8 | 279 (23.0) | 209 (24.7) | 70 (19.0) |
| Unknown | 6 (0.5) | 4 (0.5) | 2 (0.5) |
| In-hospital outcome |  |  |  |
| Survived | 1188 (97.9) | 830 (98.1) | 358 (97.3) |
| Died | 24 (2.0) | 14 (1.7) | 10 (2.7) |
| Unknown | 2 (0.2) | 2 (0.2) | 0 (0.0) |

ICU – intensive care unit

^a^Common admission diagnoses among respiratory cases were LRTI (655/846, 77.4%), sepsis (134/846, 15.8%), and bronchitis/bronchiolitis (103/846, 12.2%)

^b^Common admission diagnoses among non-respiratory cases were diarrhea (194/368, 52.7%) and febrile seizures (51/368, 13.9%)

**Supplementary table 2.** Pathogen-specific prevalence in cases and controls among infants aged <1 years, South Africa, November 2016 – October 2018

| Pathogen | Detection rate – Controls  n (%) | Detection rate – All Cases  n (%) | p-value^b,c^ | Detection rate – Respiratory Cases  n (%) | p-value^b,d^ | Detection rate – Non-respiratory Cases  n (%) | p-value^b,e^ |
| --- | --- | --- | --- | --- | --- | --- | --- |
| **Nasopharyngeal specimens** | **N=621** | **N=1107** |  | **N=780** |  | **N=327** |  |
| Influenza^a^ | 8 (1.3) | 39 (3.5) | 0.006 | 33 (4.2) | **0.001** | 6 (1.8) | 0.507 |
| Influenza A | 4 (0.6) | 24 (2.2) | 0.016 | 20 (2.6) | 0.006 | 4 (1.2) | 0.354 |
| Influenza B | 4 (0.6) | 15 (1.4) | 0.174 | 13 (1.7) | 0.082 | 2 (0.6) | 0.952 |
| Influenza C | 2 (0.3) | 8 (0.7) | 0.292 | 5 (0.6) | 0.400 | 3 (0.9) | 0.229 |
| Respiratory Syncytial Virus | 20 (3.2) | 246 (22.2) | **<0.001** | 229 (29.4) | **<0.001** | 17 (5.2) | 0.135 |
| Rhinovirus | 210 (33.8) | 359 (32.4) | 0.556 | 252 (32.3) | 0.551 | 107 (32.7) | 0.734 |
| Coronavirus 229E | 1 (0.2) | 3 (0.3) | 0.648 | 1 (0.1) | 0.872 | 2 (0.6) | 0.240 |
| Coronavirus 43 | 16 (2.6) | 23 (2.1) | 0.503 | 16 (2.1) | 0.513 | 7 (2.1) | 0.678 |
| Coronavirus 63 | 12 (1.9) | 22 (2.0) | 0.937 | 13 (1.7) | 0.709 | 9 (2.8) | 0.415 |
| Coronavirus HKU1 | 10 (1.6) | 9 (0.8) | 0.127 | 3 (0.4) | 0.017 | 6 (1.8) | 0.799 |
| Parainfluenza virus 1 | 2 (0.3) | 21 (1.9) | 0.006 | 15 (1.9) | 0.007 | 6 (1.8) | 0.016 |
| Parainfluenza virus 2 | 1 (0.2) | 14 (1.3) | 0.018 | 9 (1.2) | 0.028 | 5 (1.5) | 0.012 |
| Parainfluenza virus 3 | 15 (2.4) | 47 (4.3) | 0.050 | 37 (4.7) | 0.022 | 10 (3.1) | 0.557 |
| Parainfluenza virus 4 | 14 (2.3) | 16 (1.5) | 0.217 | 12 (1.5) | 0.324 | 4 (1.2) | 0.269 |
| Human bocavirus | 18 (2.9) | 41 (3.7) | 0.377 | 29 (3.7) | 0.397 | 12 (3.7) | 0.519 |
| Human metapneumovirus | 13 (2.1) | 51 (4.6) | 0.008 | 43 (5.5) | **0.001** | 8 (2.5) | 0.725 |
| Enterovirus | 36 (5.8) | 70 (6.3) | 0.662 | 52 (6.7) | 0.505 | 18 (5.5) | 0.853 |
| Adenovirus | 53 (8.5) | 116 (10.5) | 0.192 | 85 (10.9) | 0.140 | 31 (9.5) | 0.626 |
| *Bordetella pertussis* | 7 (1.1) | 27 (2.4) | 0.060 | 24 (3.1) | 0.014 | 3 (0.9) | 0.764 |
| *Moraxella cattarhalis* | 264 (42.5) | 402 (36.3) | 0.011 | 266 (34.1) | 0.001 | 136 (41.6) | 0.785 |
| *Chlamydia pneumoniae* | 5 (0.8) | 4 (0.4) | 0.219 | 2 (0.3) | 0.148 | 2 (0.6) | 0.741 |
| *Legionella pneumophila/longbeachae* | 0 (0.0) | 3 (0.3) | 0.194 | 3 (0.4) | 0.122 | 0 (0.0) | - |
| *Salmonella species* | 5 (0.8) | 6 (0.5) | 0.509 | 4 (0.5) | 0.496 | 2 (0.6) | 0.741 |
| *Haemophilus influenzae* | 187 (30.1) | 366 (33.1) | 0.207 | 277 (35.5) | 0.033 | 89 (27.2) | 0.351 |
| *Haemophilus influenza type b* | 5 (0.8) | 6 (0.5) | 0.509 | 5 (0.6) | 0.717 | 1 (0.3) | 0.357 |
| *Staphylococcus aureus* | 169 (27.2) | 255 (23.0) | 0.053 | 180 (23.1) | 0.075 | 75 (22.9) | 0.152 |
| *Streptococcus pneumoniae* | 300 (48.3) | 460 (41.6) | 0.007 | 311 (39.9) | 0.002 | 149 (45.6) | 0.421 |
| *Klebsiella pneumoniae* | 53 (8.5) | 152 (13.7) | **0.001** | 102 (13.1) | 0.007 | 50 (15.3) | **0.001** |
| *Mycoplasma pneumoniae* | 3 (0.5) | 10 (0.9) | 0.332 | 6 (0.8) | 0.505 | 4 (1.2) | 0.206 |
| *Pneumocystis jirovecii* | 28 (4.5) | 56 (5.1) | 0.610 | 48 (6.2) | 0.177 | 8 (2.5) | 0.114 |
| **Blood specimens** | **N=606** | **N=1032** |  | **N=722** |  | **N=310** |  |
| *Haemophilus influenzae* | 9 (1.5) | 16 (1.6) | 0.917 | 14 (1.9) | 0.528 | 2 (0.7) | 0.269 |
| *Streptococcus pneumoniae* | 16 (2.6) | 22 (2.1) | 0.509 | 12 (1.7) | 0.216 | 10 (3.2) | 0.614 |
| *Klebsiella pneumoniae* | 30 (5.0) | 50 (4.8) | 0.924 | 37 (5.1) | 0.885 | 13 (4.2) | 0.608 |
| *Pseudomonas aeruginosa* | 1 (0.2) | 2 (0.2) | 0.895 | 2 (0.3) | 0.669 | 0 (0.0) | 0.474 |
| *Streptococcus agalactiae* | 1 (0.2) | 5 (0.5) | 0.301 | 4 (0.6) | 0.249 | 1 (0.3) | 0.629 |
| *Listeria monocytogenes* | 0 (0.0) | 0 (0.0) | - | 0 (0.0) | - | 0 (0.0) | - |
| *Chlamydia trachomatis* | 0 (0.0) | 9 (0.9) | 0.021 | 8 (1.1) | 0.009 | 1 (0.3) | 0.162 |
| *Staphylococcus aureus* | 12 (2.0) | 21 (2.0) | 0.939 | 17 (2.4) | 0.642 | 4 (1.3) | 0.451 |
| *Escherichia coli* | 64 (10.6) | 105 (10.2) | 0.804 | 67 (9.3) | 0.435 | 38 (12.3) | 0.440 |
| *Ureaplasma species* | 2 (0.3) | 2 (0.2) | 0.590 | 1 (0.1) | 0.464 | 1 (0.3) | 0.985 |
| Cytomegalovirus | 184 (30.4) | 314 (30.4) | 0.979 | 227 (31.4) | 0.672 | 87 (28.1) | 0.471 |

^a^ Influenza A and influenza B combined

^b^ Significance evaluated at p<0.002 for nasopharyngeal specimens (n=30 pairwise comparisons) and p<0.005 for blood specimens (n=11 pairwise comparisons) after Bonferroni correction.

^c^ Bold font indicates p-values where the detection rate in all cases was significantly higher than in controls

^d^ Bold font indicates p-values where the detection rate in respiratory cases was significantly higher than in controls

^e^ Bold font indicates p-values where the detection rate in non-respiratory cases was significantly higher than in controls

**Supplementary table 3.** Factors associated with hospitalisation among infants aged <1 year with a respiratory admission diagnosis, South Africa, November 2016 – October 2018

| Variable | Categories | Respiratory cases  n/N  (row %) | Odds ratio^h^  (95% CI) | p-value | Adjusted OR^h^ (95% CI) | p-value |
| --- | --- | --- | --- | --- | --- | --- |
| Age group  (months) | <3 | 335/558 (60.0) | 1.2 (0.9-1.6) | 0.154 | 1.3 (1.0-1.8) | 0.070 |
|  | 3 - <6 | 201/402 (50.0) | 0.8 (0.6-1.1) | 0.128 | 0.7 (0.5-0.9) | **0.019** |
|  | 6 - <12 | 244/441 (55.3) | Ref | - | Ref | - |
| HIV status | HUU | 441/758 (58.2) | Ref | - | Ref | - |
|  | HEU | 290/449 (64.6) | 1.3 (1.0-1.7) | 0.041 | 1.5 (1.1-2.0) | **0.009** |
|  | ILWH | 33/37 (89.2) | 5.6 (1.9-16.7) | 0.002 | 6.6 (2.1-20.5) | **0.001** |
|  | Unknown | 16/157 (10.2) | 0.0 (0.0-0.0) | <0.001 | 0.0 (0.0-0.0) | **<0.001** |
| Sex | Male | 445/747 (59.6) | 1.3 (1.1-1.7) | 0.007 |  |  |
|  | Female | 335/651 (51.5) | Ref | **-** |  |  |
|  | Unknown | 0/3 (0.0) | - | - |  |  |
| Season | Summer | 178/292 (61.0) | Ref | - |  |  |
|  | Autumn | 243/422 (57.6) | 0.9 (0.7-1.3) | 0.691 |  |  |
|  | Winter | 184/328 (56.1) | 0.9 (0.7-1.3) | 0.599 |  |  |
|  | Spring | 175/359 (48.8) | 0.7 (0.5-0.9) | 0.014 |  |  |
| Underlying condition^a^ | No | 757/1372 (55.2) | Ref | - | Ref | - |
|  | Yes | 23/26 (88.5) | 4.7 (1.4-15.8) | 0.013 | 4.8 (1.3-17.6) | **0.017** |
|  | Unknown | 0/3 (0.0) | - | - | - | - |
| Malnutrition^b^ | No | 574/1140 (50.4) | Ref | - | Ref | - |
|  | Yes | 205/252 (81.4) | 4.6 (3.3-6.5) | <0.001 | 6.0 (4.0 -8.9) | **<0.001** |
|  | Unknown | 1/9 (11.1) | 0.1 (0.0-0.8) | 0.034 | 0.2 (0.0-1.7) | 0.125 |
| Feeding type | Exclusive breastfeeding | 451/850 (53.1) | Ref | - |  |  |
|  | Mixed feeding | 135/251 (53.8) | 1.1 (0.8-1.5) | 0.521 |  |  |
|  | Formula feeding | 175/267 (65.5) | 1.6 (1.2-2.1) | 0.002 |  |  |
|  | Unknown | 19/33 (57.6) | 1.2 (0.6-2.4) | 0.625 |  |  |
| Prematurity^c^ | No | 621/1183 (52.5) | Ref | - |  |  |
|  | Yes | 159/215 (74.0) | 2.5 (1.8-3.5) | <0.001 |  |  |
|  | Unknown | 0/3 (0.0) | - | - |  |  |
| Birthweight^d^ | Normal | 564/1076 (52.4) | Ref | - |  |  |
|  | Low | 182/264 (68.9) | 1.9 (1.4-2.6) | <0.001 |  |  |
|  | Unknown | 34/61 (55.7) | 1.2 (0.7-2.0) | 0.548 |  |  |
| Vaccination^e^ | No full coverage | 212/374 (56.7) | Ref | - |  |  |
|  | Full coverage | 544/988 (55.1) | 1.0 (0.8-1.3) | 0.981 |  |  |
|  | Unknown | 24/39 (61.5) | 1.3 (0.7-2.6) | 0.455 |  |  |
| Mothers/ caregivers education level | None/Primary | 364/686 (53.1) | Ref | - |  |  |
|  | Secondary/Tertiary | 409/698 (58.6) | 1.1 (0.8-1.3) | 0.636 |  |  |
|  | Unknown | 7/17 (41.2) | 0.5 (0.2-1.4) | 0.214 |  |  |
| RSV | No | 551/1152 (47.8) | Ref | - | Ref | - |
|  | Yes | 229/249 (92.0) | 12.5 (7.7-20.1) | <0.001 | 19.7 (11.4-34.1) | **<0.001** |
| Influenza | No | 747/1360 (54.9) | Ref | - | Ref | - |
|  | Yes | 33/41 (80.5) | 4.1 (1.9-9.0) | <0.001 | 5.7 (2.3-14.1) | **<0.001** |
| Human metapneumovirus | No | 737/1345 (54.8) | Ref | - | Ref | - |
|  | Yes | 43/56 (76.8) | 3.0 (1.6-5.6) | 0.001 | 4.1 (2.0-8.6) | **<0.001** |
| *Klebsiella pneumoniae^i^* | No | 678/1246 (54.4) | Ref | - |  |  |
|  | Yes | 102/155 (65.8) | 1.7 (1.2-2.5) | 0.003 |  |  |

^a^Underlying condition includes any of the following: asthma, chronic lung, heart, liver or renal disease, stroke, sinusitis, organ transplant, anaemia, immunosuppressive therapy, splenectomy, diabetes, burns immunoglobulin deficiency, autoimmune disease, nephrotic syndrome, cancer, spinal cord injury, seizure disorder, cerebral palsy, congenital heart disease, other congenital disorder, obesity or chronic gastrointestinal problems.

^b^Malnutrition defined as a weight-for-age <-2 standard deviations from the WHO mean Z-score.

^c^Prematurity defined as gestational age at birth of <37 weeks.

^d^Low infant birthweight defined as <2500g.

^e^Vaccination defined as full vaccine coverage for age, using the *Haemophilus influenzae* type b vaccine given as part of the routine infant immunisation schedule at 6,10, 14 weeks as a proxy

^f^Admission diagnosis: respiratory diagnosis includes apnoea, neonatal sepsis, bronchiolitis, pneumonia, tuberculosis and bronchitis) and non-respiratory diagnosis includes encephalitis, viral illness, diarrhoea, febrile seizures, meningitis, sepsis (non-neonatal) and other diagnosis.

^g^Chi-squared test p-value

^h^Mixed effects regression model, accounting for clustering by site

^I^ *Klebsiella pneumoniae* detected in nasopharyngeal specimens

**Supplementary table 4.** Serological influenza attack rate^a^ among infants aged <1 year, South Africa, November 2016 – October 2018

| Influenza subtype/lineage | Attack rate n/N (%)* | | | | |
| --- | --- | --- | --- | --- | --- |
|  | Controls | Respiratory cases | p-value^b^ | Non-respiratory cases | p-value^c^ |
| Any influenza (A/B) | 51/370 (13.8) | 65/378 (17.2) | 0.197 | 19/163 (11.7) | 0.503 |
| Influenza A^d^ | 30/370 (8.1) | 22/378 (5.8) | 0.219 | 6/163 (3.7) | 0.061 |
| Influenza A(H1N1)pdm09 | 10/370 (2.7) | 13/382 (3.4) | 0.577 | 3/163 (1.8) | 0.546 |
| Influenza A(H3N2) | 21/371 (5.7) | 11/381 (2.9) | 0.060 | 3/163 (1.8) | 0.050 |
| Influenza A(H3N2) – 2017 only^f^ | 15/241 (6.2) | 8/231 (3.5) | 0.164 | 1/98 (1.0) | 0.041 |
| Influenza A(H1N1)pdm09 – 2018 only^f^ | 6/125 (4.8) | 9/145 (6.2) | 0.615 | 2/62 (3.2) | 0.617 |
| Influenza B^e^ | 17/365 (4.7) | 22/356 (6.5) | 0.290 | 5/153 (3.3) | 0.474 |
| Influenza B/Victoria | 8/368 (2.2) | 10/357 (2.8) | 0.587 | 2/152 (1.3) | 0.517 |
| Influenza B/Yamagata | 12/364 (3.3) | 17/356 (4.8) | 0.313 | 3/154 (2.0) | 0.403 |

^a^Attack rates were calculated as the number of individuals that seroconverted (≥4-fold rise in antibody titre) divided by the number of individuals with paired sera tested (follow up blood sample collected >13 days after enrollment blood sample)

^b^Chi-squared test for respiratory cases compared to controls

^c^Chi-squared test for non-respiratory cases compared to controls

^d^Seroconverted to A(H1N1)pdm09 and/or A(H3N2)

^e^Seroconverted to B/Victoria and/or B/Yamagata

^f^Serological attack rate calculated for the dominant circulating subtype in the influenza season of the specific year. In 2017 and 2018 influenza A(H3N2) and A(H1N1)pdm09 were the dominant subtypes in South Africa, respectively, as determined by sentinel syndromic surveillance.

**Supplementary table 5.** Proportion of infants aged >1 year with haemagglutination inhibition (HAI) titres (≥1:40) at enrollment, South Africa, November 2016 – October 2018

| Influenza subtype/lineage | Proportion n (%)* | | |
| --- | --- | --- | --- |
|  | Controls  N=575 | Respiratory cases  N=737 | Non-respiratory cases  N=325 |
| Influenza A(H1N1)pdm09 | 50 (8.7) | 86 (11.7) | 33 (10.2) |
| Influenza A(H3N2) | 98 (17.0) | 134 (18.2) | 49 (15.1) |
| Influenza B/Victoria | 29 (5.0) | 84 (11.4) | 42 (12.9) |
| Influenza B/Yamagata | 102 (17.7) | 141 (19.1) | 43 (13.2) |

**Supplementary figure 1.** Number of infants enrolled in the Infant Burden Study by year and month, South Africa, November 2016 – October 2018 (N=1923)

**Supplementary figure 2.** Percentage of infants enrolled in the Infant Burden Study by study year, case definition and age group, South Africa, November 2016 – October 2018 (N=1923)
